# A polyphagous, tropical insect herbivore shows strong seasonality in age-structure and longevity independent of temperature and host availability

**DOI:** 10.1038/s41598-021-90960-7

**Published:** 2021-06-01

**Authors:** Mst Shahrima Tasnin, Michael Bode, Katharina Merkel, Anthony R. Clarke

**Affiliations:** 1 School of Biology and Environmental Science, Queensland University of Technology (QUT), Brisbane, QLD 4001 Australia; 2 School of Mathematical Sciences, Queensland University of Technology (QUT), Brisbane, QLD 4001 Australia

**Keywords:** Ecology, Ecology

## Abstract

*Bactrocera tryoni* is a polyphagous fruit fly that is predicated to have continuous breeding in tropical and subtropical Australia as temperature and hosts are not limiting. Nevertheless, in both rainforest and tropical agricultural systems, the fly shows a distinct seasonal phenology pattern with an autumn decline and a spring emergence. Temperature based population models have limited predictive capacity for this species and so the driver(s) for the observed phenology patterns are unknown. Using a demographic approach, we studied the age-structure of *B. tryoni* populations in subtropical Australia in an agricultural system, with a focus on times of the year when marked changes in population abundance occur. We found that the age-structure of the population varied with season: summer and autumn populations were composed of mixed-age flies, while late-winter and early-spring populations were composed of old to very old individuals. When held at a constant temperature, the longevity of adult reference cohorts (obtained from field infested fruits) also showed strong seasonality; the adults of spring and early autumn populations were short-lived, while late autumn and late winter adults were long-lived. While still expressing in modified landscapes, the data strongly suggests that *B. tryoni* has an endogenous mechanism which would have allowed it to cope with changes in the breeding resources available in its endemic monsoonal rainforest habitat, when fruits would have been abundant in the late spring and summer (wet season), and rare or absent during late autumn and winter (dry season).

## Introduction

Approximately 75% of tropical forests demonstrate strong seasonality^1^, and within those forests some tropical insects also show predictable phenology patterns^2,3^. Seasonal fluctuation of abundance of tropical herbivorous insects has been linked to monsoonal cycles of wet and dry seasons^4,5^, with an increased number of insects in the wet season considered to be related to the increased availability of new leaf and vegetative material for feeding and breeding^6–8^. For example, the abundance of Ithoniine butterflies increases with the onset of the wet season and declines dramatically during the dry season, due to the availability, or lack thereof, of host plants for oviposition and caterpillar feeding^4^. An exception to this pattern is thought to exist for polyphagous herbivore species where hosts are assumed to be available throughout the year: in this case continuous breeding is predicted as both temperature and hosts are not limiting^9–11^. However, as discussed further below, even for polyphagous species resources may be seasonally lacking.

While numerous studies on the phenology of tropical insects have investigated the drivers of changing population abundance, e.g. rainfall, host availability, etc.^12–14^, we are not aware of any study which has gone further and investigated if changes in population abundance are also associated with changes in population demography, for example birth rates and death rates. Thus, the demographic processes behind seasonal population change remains unknown in tropical insects^2^.

The polyphagous tropical fruit flies (*Bactrocera* spp., Diptera: Tephritidae) include invasive pest species for which modelers and biosecurity risk analysts have made explicit assumptions that breeding is, or can be, continuous in the tropics because hosts and temperature are not limiting^11,15–17^. However, as breeders in the fruits of tropical forests^18^, this assumption may be incorrect as the potential hosts in rainforest may be seasonally restricted because of monsoon driven flowering and fruiting^19^. Thus, the flies may also have seasonally changing demographics associated with these events but, if so, this is unknown as it is for other seasonally impacted tropical insects.

Demographic study of an insect can provide vital information about the basic properties of an insect population^20^ as it deals with four major aspects of a population: size, distribution, structure and change, where structure means the distribution of a population by age and sex and change implies total growth or decline of the population^21,22^. The conventional methods of demographic study involve life-table techniques, mortality models, and population comparison^23^. For insects, whose small size makes it highly difficult to track individuals in the field, demographic data is generally obtained using life-table techniques which are dependent on the use of mortality data from known age individuals maintained in the laboratory^24^, or through capture-recapture methods to assess aging in the wild^25^.

While it is recognized that there is growing importance in understanding aging in the wild^26,27^, it can be very difficult to accurately estimate the age of wild insect populations using conventional age grading methods^28,29^. Developed as an extension of the life-table approach to age wild insect populations^26,30^, the demographic method uses combined information from a ‘captive cohort’ (individuals captured from the wild at an unknown age whose post-capture longevity is then recorded under standard conditions) and a ‘reference cohort’ (adult individuals emerging from wild collected juveniles [e.g. larvae or caterpillars], then held under the same standard conditions to provide whole-of-life longevity) to back calculate the age-distribution of the unknown age wild adults through the deconvolution method^28,31^. The approach is based on the concept that if individuals can be captured from a wild population without bias to their proportional age-distribution in the wild, and the force of mortality is only dependent on age and independent of rearing environment, then the age-distribution in the wild is equal to the death-distribution in captivity and so the former can be predicted by the later^31,32^. The demographic approach has been implemented to explore population demography over seasons in a limited number of temperate and Mediterranean insects^28,33–35^, but has not been applied to tropical insects.

In this paper we explore the changes in demographic structure of a tropical insect, using as our test animal *Bactrocera tryoni* (Froggatt) (Diptera: Tephritidae), a native multivoltine Australian insect originally endemic to the Australian east coast tropical and subtropical rainforests^16,36^, but which since the late 1800s has also become a pest of horticulture^37^. The Australian east coast rainforests have a restricted flowering and fruiting season which occurs during austral spring and early summer, initiated by the start of the wet-season monsoon^38^. In temperate parts of Australia, where *B. tryoni* is invasive^39^, its breeding is considered temperature limited, with a winter decline in population numbers^40,41^. However, paradoxically, this species also shows “overwintering” population declines in subtropical and tropical Australia in both endemic rainforest^42^ and man-made habitats^43,44^, where lower threshold temperatures are not limiting^45,46^. A simple absence of suitable hosts during this population depression period in the tropics also does not explain the phenology of the fly, as a recent phenological study demonstrated little correlation of either temperature or host fruit availability on the population phenology of *B. tryoni* at an agricultural subtropical site^47^.

Using the demographic approach^28,31^, we initiated a study of age-structure changes of *B. tryoni* populations in relation to phenological changes of those populations to better understand the species’ demographic ecology in its tropical range. Ideally our work would have be done in rainforest, but *B. tryoni* occurs at low abundance in rainforest^48^, as do other fruit flies^49^, and our methodology required large numbers of flies and larval infested fruit which were not obtainable from local rainforest. Fortunately, the phenology pattern on *B. tryoni* in the study region is identical whether in rainforest or agricultural landscapes^42–44^. Thus, for logistic reasons and because we believed it would not impact on our results, we worked in an agricultural situation where flies and fruit were highly abundant. As the core element of the demographic approach, we collected captive and reference cohorts of *B. tryoni* during different seasons. The study addressed three major questions: i. At an agricultural, subtropical site where host fruits are always available, does the tropical insect *B. tryoni* breed continuously through the year, or is there demographic evidence for gaps in breeding? ii. Does the demographic structure of field populations change over season? iii. Can the demographic approach help explain the “winter” decline in a region where lower temperatures are not limiting? Additional to its value for understanding this particular species, we believe the demographic methodology we modify and refine here can be applied to other tropical insects for which demographic studies are absent.

## Results

A total of 901 captive males and 242 reference males were studied across all sampling occasions. The survival function of reference males was significantly different from the captive males (χ^2^ = 179.52, Df = 1, *P* < 0.001). The mean (and median) longevity of reference males was 74.8 days (and 57 days), while for captive males the mean (and median) post-capture longevity was 28.8 days (and 19 days). Eleven reference males (4.5%) lived > 200 days, with a maximum longevity of 253 days; while for captive males just a single male lived > 200 days, dying at 229 days.

### Seasonal influence on the longevity of reference flies

**Figure 1:**
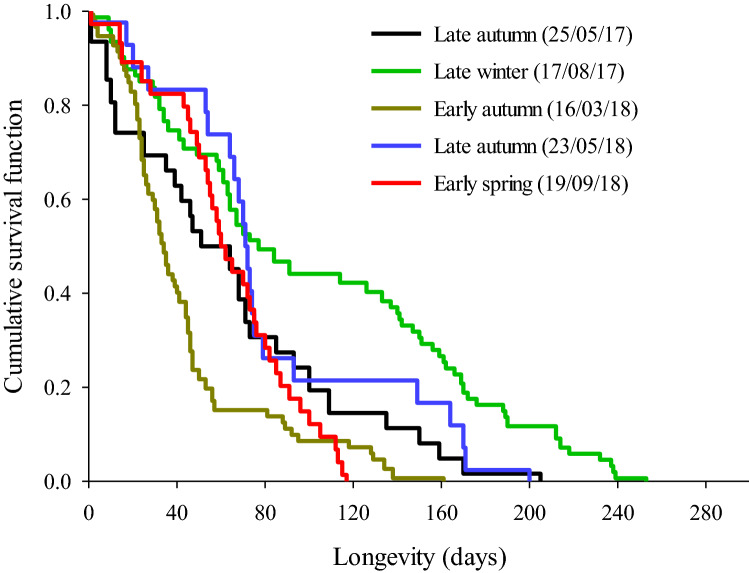
Cumulative survival curves of five cohorts of adult male *Bactrocera tryoni* that emerged in the laboratory from field infested fruit (= reference cohorts) collected during different seasons in 2017–18 from a site in subtropical Australia. In the laboratory flies were held at 27 °C constant temperature and had ad libitum access to water and food.

The reference males emerging in the laboratory from field infested fruits collected at five time points during 2017 and 2018 exhibited significant differences in survival probability in pairwise log-rank tests (Table 1), despite being held under the same constant conditions from emergence. The late-winter 2017 males were long-lived, with more than 45% living longer than 120 days and the longest living 253 days. The survival probability of late winter 2017 males was significantly greater than other seasons except late autumn 2018 (Df = 1 and *P* = 0.005; < 0.001; 0.218 and < 0.001 in pairwise comparisons between late winter 2017 vs. late autumn 2017; early autumn 2018; late autumn 2018; and early spring 2018 respectively). In contrast, early-autumn 2018 males were short-lived, with only 6% living beyond 120 days and the longest-lived dying at 161 days. The survival probability of early-autumn 2018 males was significantly lower from all other seasons (Df = 1 and *P* = 0.009; < 0.001; < 0.001; and 0.025 in pairwise comparisons between early autumn 2018 vs. late autumn 2017; late winter 2017; late autumn 2018 and early spring 2018, respectively). The survival probability of late-autumn 2017, late-autumn 2018, and early-spring 2018 males were intermediate between these two extremes. While male survival of late-autumn 2017, late-autumn 2018 and early spring 2018 cohorts did not significantly differ from each other, the early spring male cohort had a steeper death curve at the end of the population and no males lived longer than 120 days. In contrast, 22–25% of the late-autumn population lived longer than 120 days (Fig. 1).

**Table 1:** A pairwise comparison of survival function of five cohorts of adult male *Bactrocera tryoni* that emerged in the laboratory from field infested fruit (= reference cohorts) collected during different seasons in 2017–18 from a site in subtropical Australia.

Seasons and dates	Fruit type	No. of males	Late winter 17/08/17		Early autumn 16/03/18		Late autumn 23/05/18		Early spring 19/09/18	
			χ^2^	*P*	χ^2^	*P*	χ^2^	*P*	χ^2^	*P*
Late autumn 24/05/17	Custard apple	31	8.07	**0.005**	6.822	**0.009**	0.939	0.333	0.642	0.423
Late winter 17/08/17	Custard apple	77			44.366	**< 0.001**	1.519	0.218	14.504	**< 0.001**
Early autumn 16/03/18	Carambola	76					14.955	**< 0.001**	4.991	**0.025**
Late autumn 23/05/18	Custard apple	21							3.353	0.067
Early spring 19/09/18	Carambola	37								

Bold values indicate significant difference at *P* < 0.05.

In the laboratory flies were held at 27 °C constant temperature and had ad libitum access to water and food.

**Figure 2:**
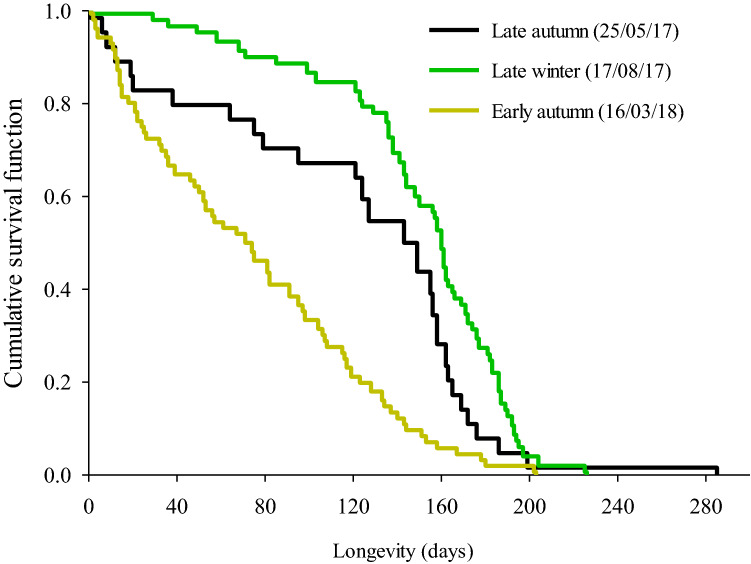
Cumulative survival curves of three cohorts of adult female *Bactrocera tryoni* that emerged in the laboratory from field infested fruit collected during different seasons in 2017–18 from a site in subtropical Australia. In the laboratory flies were held at 27 °C constant temperature and had ad libitum access to water and food.

The reference females emerged from infested host fruits collected during three different seasons also showed significant difference in survival probability. Similar to males, the late autumn 2017 and late winter 2017 females were long-lived while early autumn 2018 females were short-lived (Chi^2^ = 12.306, Df = 1, *P* < 0.001 and Chi^2^ = 56.012, Df = 1, *P* < 0.001 in a pairwise comparisons between early autumn 2018 vs. late autumn 2017 and early autumn 2018 vs. late winter 2017 respectively). However, late autumn 2017 and late winter 2017 seasons survival probability did not significantly differ (Chi^2^ = 3.164, Df = 1, *P* = 0.08) (Fig. 2).

### Survival of captive males

Mean and median longevity of captive males showed seasonal variation. Mean and median post-capture longevity was highest during early autumn 2017 at 41.6 and 34 days, respectively, with longevity then declining through the late autumn and reaching a minimum in late winter and early spring. The mean and median longevity started to increase again in early summer until peaking again at around 39 days in early autumn 2018 (Table 2).

**Table 2:** Survival of wild *Bactrocera tryoni* males (= captive cohorts) in the laboratory.

Sampling season	Date of trapping	Orchard’s type	No of males studied	Mean post-capture longevity (days)	Median post-capture longevity (days)	Maximum Longevity (days)
Early autumn	13/03/2017	Persimmon, Loquat	50	41.6	34	163
Late autumn	24/05/2017	Guava, Custard apple	334	29.9	19	141
Late winter	17/08/2017	Mulberry	126	11.4	6	229
Early spring	7/09/2017	Custard apple	41	16.2	5	120
Early summer	17/11/2017	White sapote	126	30.4	18	169
Early autumn	16/03/2018	Carambola	178	39.8	38	152
Early spring	19/09/2018	Avocado	50	18.7	11.5	135

Flies were collected as adults during different seasons in 2017–18 from a site in subtropical Australia. In the laboratory flies were held at 27 °C constant temperature and had ad libitum access to water and food.

### Age-structure of wild population of *B. tryoni*

#### Age-structure estimation and consistency

**Figure 3:**
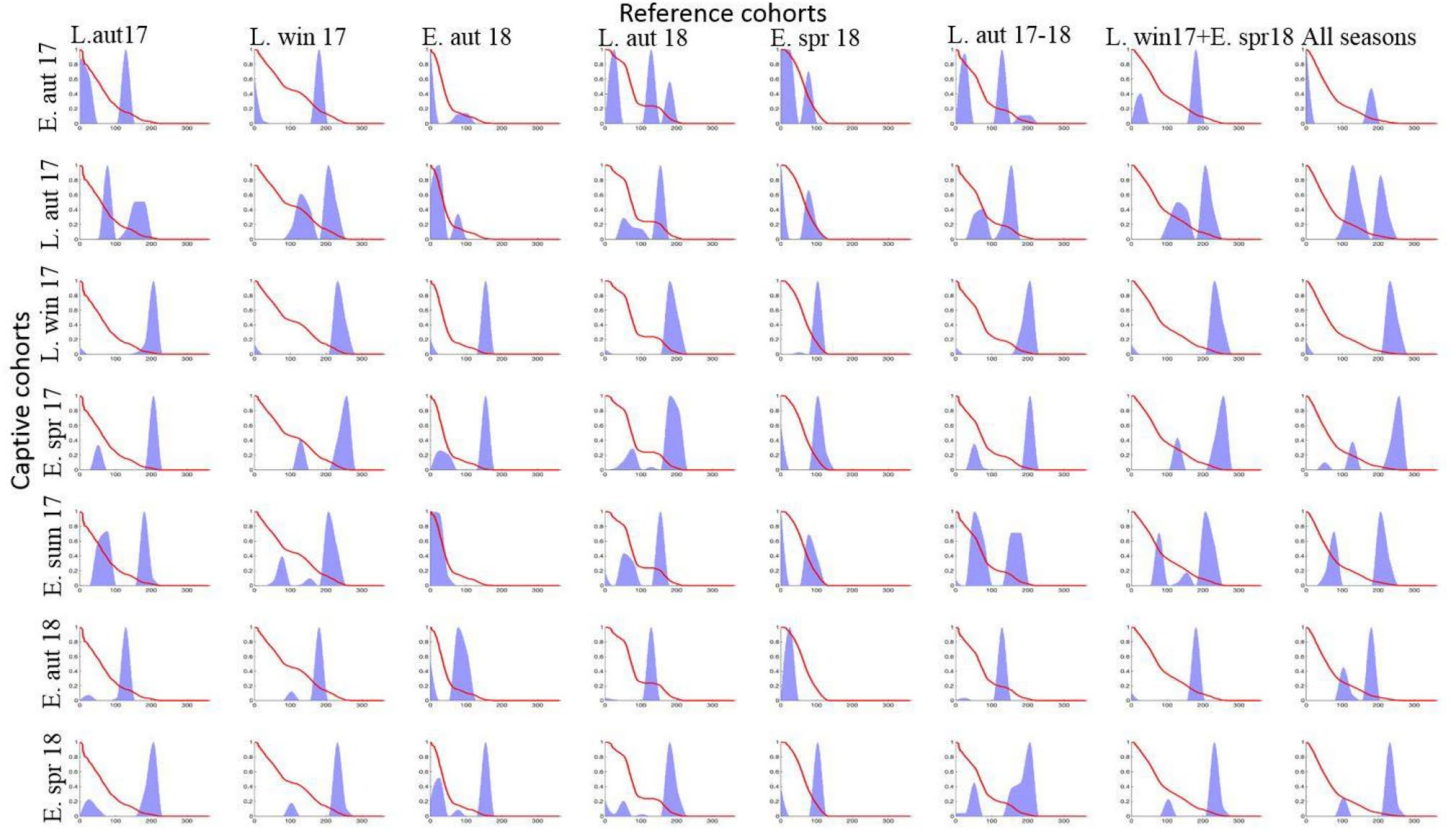
Probability density of age (in days) of wild *Bactrocera tryoni* captured during different seasons in 2017–18 from a site in subtropical Australia. Each graph is the fitted age-distribution for the season as estimated using the likelihood function with all possible combinations of reference cohorts. The red line is the survival function of the reference cohort used in the estimation of the likelihood function and the blue curves are age-distribution (L = late, E = early, aut = autumn, win = winter, spr = spring, sum = summer, 17 = 2017 and 18 = 2018).

While the best fit model for each captive cohort was calculated (Suppl. Table 1), we found that applying different reference cohorts to the same captive cohort produced estimated age-structure predictions that were qualitatively consistent, but not quantitatively consistent (Fig. 3). The reason for this quantitative inconsistency was because of the survival variation in the reference cohorts. Thus, we focus on presenting the results in qualitative terms (i.e. young, middle-aged, and old) which are generally highly consistent regardless of reference cohort used, and as such gives us confidence in the robustness of the results. We note that in some seasons, notably the autumn samples, the application of different control cohorts to the same captive cohort provided qualitatively different outcomes as well as quantitatively different outcomes: we discuss such cases individually.

#### General pattern of age-distribution

The age-structure of wild *B. tryoni* populations generated by the likelihood method showed that the demographic structure of the population varied with season. For all seasons the population never consisted of more than three age-groups of individuals. The early-autumn sampling, which had the maximum number of age-groups, consisted of a mixed-age population with young, middle-aged and old individuals. The late-autumn population consisted of two age-groups of middle-aged and old flies, while the late winter and early spring populations were dominated by very old individuals. However, by early summer, a mix of ages was again apparent with young, middle-aged and older flies. More detail on each season follows.

Early autumn 2017 and 2018: The early autumn 2017 population consisted of a mix of two to three age-groups of flies. The most common pattern showed only two age-groups, one young and the other either middle-aged or old (Fig. 3). The model estimation for the same season in 2018 showed a different age-distribution pattern, with the population containing a single age cohort of middle-aged to old age flies and only a few very young flies. Despite the apparent differences between the years, a pairwise long-rank test of the survival functions of early autumn 2017 and 2018 populations found no significant difference between these two collections (χ^2^ = 0.4, Df = 1, *P* = 0.54).

Late autumn 2017: The age-distribution pattern was highly consistent across the models and showed two dominant age-groups of individuals consisting of middle-aged to old males. Two of the eight models also estimated the presence of some young flies (Fig. 3).

Late winter 2017: The age-structure predictions across models were again highly consistent, showing that the population contained a single age-group of predominantly old flies. Some models predicted a small number of very young individuals, but middle-aged flies were entirely absent from all models (Fig. 3).

Early spring 2017 and 2018: The early spring populations from 2017 and 2018 again showed consistency in age-structure, with the common pattern being that the populations contained one dominant age-group of predominantly very old flies, and a second small group of young individuals (Fig. 3). The log-rank test comparing the survival functions of the 2017 and 2018 early spring captive cohorts did not detect a significant difference (χ^2^ = 0.959, Df = 1, *P* = 0.33).

Early summer 2017: As for the early autumn populations, the early summer population consisting of a mixed age-group with young, middle age and moderately old age flies. As for the early autumn cohorts, the models run with different control cohorts on the early summer captive cohort gave a greater range of both qualitative and quantitative predictions, possibly due to the greater range of fly ages in the field (Fig. 3).

## Discussion

Contrary to expectations based on population modelling^10,16^, the population of *B. tryoni* at our site during the year was composed of only one or two, very occasionally three, generational age-groups. The presence of old age individuals in late winter and early spring very clearly indicates a gap in population growth from mid-autumn to late winter. The presence of some young individuals in the early spring indicates that the population begins breeding again in the spring and the mixed-age population during summer and early autumn is an indication of continuous breeding from spring to autumn. Notably, the “autumn to winter” breeding cessation is not temperature driven, as the lower temperatures are not cold enough to stop breeding^45,50,51^. Breeding at the site was also not halted due to lack of host fruit for larvae, as fruit are continuously available at that site^47^. The following discussion develops these points with respect to *B. tryoni*, but then broadens to examine the implications of the findings, and the methodology used, to understanding the demography of other tropical monsoonal insects.

The longevity of controlled reference flies that emerged in the laboratory from field collected infested fruits showed strong seasonality, despite being held under the same constant conditions. Early spring and early autumn flies were relatively short-live, while late-autumn and late-winter populations were very long-lived. This is unlikely to be linked to the original host fruit of the flies, as larval rearing host has little impact on the longevity of adult *B. tryoni*^52^. Similarly, while variation in insect adult lifespan can be induced by dietary variation^53,54^, and this has been demonstrated in *B. tryoni*^55^, it is not relevant in our study as flies were maintained in the laboratory, from emergence, on a standard and unchanging diet. Rather, decreasing longevity in insects is a common life-history trade-off against increasing fecundity^56,57^, and while not measured here we strongly suspect the short-lived spring population would have been significantly more fecund than the long-lived winter population. Variation in adult longevity (and possibly fecundity) in *B. tryoni* might be an epigenetic modification induced by environmental stimuli experienced during an earlier developmental stage^58^. *Bactrocera tryoni* pupae and adults have rapidly enhanced capacity to survive freezing temperatures (− 4 °C) after experiencing a period of cold acclimation in critical periods of earlier developmental stages^59^, indirect evidence for epigenetic modification of physiology in this species. Further, Kumaran, et al.^60^ have demonstrated covalent histone modifications in the genome of *B. tryoni*, i.e. evidence for the presence of an epigenome. While requiring further research to confirm, we believe the evidence points to the different longevities of the control cohorts being attributable to seasonally linked cues, acting on larvae in fruit, leading to histone modification of the genome and subsequent changes in adult longevity.

When combined, the data sets strongly suggest that *B. tryoni* has an endogenous mechanism which allows it to rapidly increase reproductive capacity during the spring/summer wet-season fruiting period of its indigenous rainforest habitat^36,38^, followed by an adult quiescence mechanism during the non-fruiting dry season. This pattern explains not just our data, but also matches the well documented population phenology of the species, which consistently shows rapid spring increases with populations peaking in late summer and early autumn, before declining again until the following spring^43,44^. The population thus effectively “resets” itself every spring to a single starting generation, rather than having the continuous breeding and overlapping generations predicted by models which assume that temperature and hosts are not limiting for this polyphagous, tropical species^10,15,16^. Further indirect evidence of the endogenous nature of this pattern comes from factory-scale mass rearing data of *B. tryoni* for the Sterile Insect Technique^61,62^. For example Dominiak, et al.^61^ showed that flies in a enviroment controlled mass-rearing factory, over several years, had reduced breeding during the winter months, had increasing longevity leading into winter, and decreasing longevity leading into summer. The authors expressed surprise at, but could not explain, a mechanism for their results.

The study showed that the demography, and not just the abundance of the tropical *B. tryoni* varied with season, and such they are not unlike temperate and Mediterranean insects^28,33,35^. The Mediterranean species *Ceratitis capitata* (Wiedemann) also has an early spring population that consists of mainly old individuals due to a lack of quality breeding hosts in the winter months. However, with the onset of summer and autumn the availability of suitable hosts increases, which results in increased population size and age-structure heterogeneity as the season progresses through to winter^28,63^. In the temperate region, *Drosophila melanogaster* Meigen similarly showed a uniformly young early spring population, which rapidly grew such that late season populations contained mixed age individuals^35^. For each of the three species, the variation in age-structure at different seasons arise from similar cycles of reproductive activity, although those activities are influenced by different seasonal effects. For the temperate *D. melanogaster* cold winter temperatures limit breeding; for *C. capitata* a lack of quality hosts during the mild, wet winter months limits breeding; and for *B. tryoni* an endogenous quiescence mechanism, that we believe to be an adaptation to surviving a monsoonal dry season lack of hosts (but still operating in a human modified landscape), resets breeding to the start of the wet season.

While restricted to a single species our data has clear implications for, and link to, other tropical insects. While it is well recognised that the availability of resources for breeding can vary greatly in the tropics due to seasonal rainfall cycles^3–5^, we present data here that suggests both a mechanism for maximising the usage of breeding hosts when they are available, and also for aiding population survival in their absence. Longevity is closely linked to reproduction, which may vary with changes in ecological environment^23^. Thus, short lifespan in tropical insects during favourable seasons is likely to be linked to a greater reproductive output in a breeding-resource rich environment to allow enhanced opportunity to take utilise those resources. On the other hand, a long lifespan is likely to be related to lower reproductive output during a period of scarcity of resources and uncertain environmental conditions^56,57^ for example the central African nymphalid *Euphaedra medon* (Linnaeus) can live for more than nine months in a mode of reproductive diapause to cope with very harsh dry seasons^64,65^. Seasonality in reproductive strategy has also been reported in several species of tropical satyrine butterflies, where reproductive activity markedly declines during the dry seasons and increses in the wet season^7^. To these butterfly studies, we now add a dipteran example, which strongly suggests that life-history trade-offs between longevity and reproducton can occur at the seasonal level and likely involve complex physiological and behavioural adaptive mechanisms in tropical insects.

Quiescence is well documented in monsoonal tropical insects and variation in temperature, rainfall, light intensity and day length can all affect diapause entry and exist of tropical insects^9,66,67^. While this study did not investigate the mechanism of the quiescence we identified, we do not believe it to be triggered by one of these standard cues. The start of the spring activity of *B. tryoni* is highly consistent along the east coast of Australia from far-north Queensland to southern New South Wales^10,42–44^, a north–south distance of approximately 2600 km. Across this range, day degree accumulation, day length and rainfall all vary substantially, so cannot be the cues triggering quiescence entry or exit. However, we note that in some Diptera there is genetic capacity in diapause initiation such that widely distributed species can received different environmental cues (i.e. day length) which still trigger the same diapause exit date^68^. Now that it is apparent that *B. tryoni* has some form of adult diapause/quiescence, which was not known when this study commenced, more targeted diapause research can be undertaken. To reveal the underlying mechanisms of this fly phenology further demographic, genetical and physiological studies are in progress.

Our study demonstrated that the likelihood function method we used here, an experimentally similar but mathematically different approach to deconvolution modelling^26,28,31^ can be applied to estimate the age of wild tropical insects qualitatively. However, the method cannot be applied for a quantitative measure unless a biologically appropriate reference cohort can be found. We found it to be biologically quite complex to determine the appropriate reference cohort for a given captive cohort, given the potential extreme longevity of flies in the field, and the endogenous variability across the reference cohorts. We thus paired each captive cohort with all the possible combination of reference cohorts to broaden our opportunity to find a general pattern. While it may be appropriate to use a combined reference cohort for temperate insects^28,35^, we do not recommend it as an approach for tropical species which may show seasonal variation in longevity as relying on a single control cohort, or combining control cohorts, may lead to bias in the predicted ages of captive cohorts. Developing better methodology for appropriatly linking captive and control cohorts is needed to improve the quantitative predictions of the demographic approach when applied to tropical insects.

While there is a conflict about the reliability of using data from the laboratory reared animals to reveal life history traits^27^, the demographic method we followed here shows that flies that were collected from the wild as an adult and then held for the rest of their life in captivity or collected as an immature and then held in the captivity for their whole adult life, can still provide novel insights into the aging of wild insects during different seasons. Thus, we consider the demographic approach of age-estimation to be not only useful to understand ageing in wild but agree with other authors that it can also be a useful tool for determining information about the reproduction, longevity, and other life history traits of wild insects^26,69,70^. Further, with refinement to better align captive and reference cohorts, we believe it has the potential to greatly advance insect demography in the tropics.

## Materials and methods

### Study site and sampling seasons

A demographic approach similar to that developed by Carey and Colleagues^28,31^ is used to estimate the age-structure of wild population of *Bactrocera tryoni.* Field work was undertaken at Tropical Fruit World (28.17′S, 153.31′E), an agro-tourism property surrounded by remnant rainforest and pasture located in the Tweed Valley, New South Wales, Australia. The site is located in the coastal hinterland, has a subtropical climate with mean summer and winter temperatures of 28 °C and 21 °C, respectively, and a mean annual rainfall of 1622 mm. The farm was established in 1983 and grows approximately five hundred varieties of mature tropical and subtropical fruit trees. The diversity of trees on the property meant that fruit suitable for *B. tryoni* breeding are available year-round. Because of the tourism aspect of the property, there is a minimal use of pesticides at the site^47^.

The captive cohorts were collected at five time points in the year of 2017: 13th Mar (early autumn), 25th May (late autumn), 17th Aug (late winter), 7th Sep (early spring), and 17th Nov (early summer). In 2018, the early autumn (16th Mar) and early spring (19th Sep) seasons were repeated. The timing of samples was chosen based on the known phenology of *B. tryoni* in subtropical Australia^43,44,47^ which covered the end of winter period when population abundance is low just before the start of the spring population increase, the start of the spring increase, periods of high population abundance during summer and early autumn, and then during the late autumn decline. Note, the term “overwintering” in this paper is used for convenience to describe the period of low fly abundance which occurs from mid-autumn through to late winter. However, by doing so, we do not wish to infer that the fly is cold-temperature limited. The site’s mean winter average of 21 °C is well above lower temperature thresholds for *B. tryoni* and two independent climatic models have each predicted six to eight generations of the fly per year at subtropical site based on day degree accumulation above a 12 °C lower threshold^15,16^.

### Sampling and rearing of wild caught captive flies (W)

Flies from the field were live captured following their attraction to BioTrap (V2 X type) baited with cue-lure (4-(4-acetoxyphenyl)-2-butanone), a commonly used attractant for surveillance and monitoring of male *B. tryoni*^37,44^ or freshly cut slices of ripe tomato, *Lycopersicon esculentum* Mill, to which males also respond^71^. On each sampling occasion, four cue-lure and four tomato-based traps were hung in fruiting orchards at 8.00 am in the morning and collected five hours later at 1.00 pm. Traps were placed approximately 10 m apart, each located in a separate tree in a fruiting orchard. Traps were rotationally observed by two to three people and attracted flies were hand captured using a 50 ml plastic vial as the flies walked on outside of the trap before entering. Thereafter, flies were immediately transferred to a white mesh cage (32 × 32 × 32 cm) containing water and sugar and kept in a shaded place until returned to the laboratory in an air-conditioned vehicle.

On return to the laboratory, on the same day of collection, flies were transferred to a constant temperature cabinet (TC). The initial TC temperature was set to mirror current maximum field temperatures, and then adjusted (up or down as required) at a rate of 1.0 °C every 8 h until it reached the standard insectary temperature of 26 ± 1 °C. This temperature ramping was done as initial trials showed that this gradual temperature adjustment minimised sudden post-capture mortality. After three days, by which time temperature adjustment was completed, flies were transferred to the insectary and separated into new holding cages as groups of 30 to 40 flies. At this point flies were visually reassessed (first assessment was done at time of capture) to confirm that only *B. tryoni* were held. The only other local fruit fly species easily confused with *B. tryoni* is *B. neohumeralis* (Hardy), and this species is rare at the study site^47^. Both species can be distinguished morphologically by the colour of humeral calli where *B. tryoni* has yellow and *B. neohumeralis* has brown humeral calli respectively^72^. Any flies that died within three days of collection (i.e. before transfer to the insectary) were excluded from post-capture longevity analysis as their death, while possibly natural, could not be separated from mortality due to handling shock. In the insectary flies were maintained at 26 ± 1 °C, 65 ± 1% RH and supplied with water, sugar and yeast hydrolysate ad libitum. *Bactrocera* species are dusk breeders^73,74^ and in an insectary need changing natural lighting conditions to achieve mating. Thus, our insectary (as with most specialist fruit fly insectaries) has large windows and artificial lighting was turned off at 5.00am each day before the onset of dusk and turned on at 7.00 am (approximately 10L:14D cycle with natural dawn and dusk). This means we could not control for the effects of changing day length in our trial.

Because of methodological constraints, it was impossible to collect female flies and all the captive cohorts consist of male flies only. Once sexually mature, males *B. tryoni* response to cue-lure and tomato was constant until 12 weeks^75^ and 15 weeks^71^ respectively. Therefore, with respect to the experimental need to sample from the field to an unbiased age estimate^70^, our sampling will not recover sexually immature males (sexual maturation occurs at approximately 10 days of age in *B. tryoni*^76^), and so the presence of very young flies will be under-estimated in our study. However, between 10 and (at least) 105 days of age, we believe the males were sampled without age bias.

### Collection of infested fruits and rearing of reference flies

Concurrent with the collection of wild adults, except for the early autumn 2017 sample, ripe host fruits available in the field were collected and returned to the laboratory. The fruits collected varied with season, but included carambola, custard apple, guava, mulberry and white sapote. In the laboratory, fruits were placed in ventilated plastic containers on a layer of vermiculite and held in an incubator at 26 °C and 65% RH. After 12 days the vermiculite was sieved and obtained pupae were placed in a mesh cage in the same insectary which held the captive flies. During early spring and early summer 2017 very few flies emerged from the fruits and so these dates were excluded from subsequent calculations. Thus, we obtained five reference cohorts that were collected on the 24^th^ May 2017 (late autumn), 17^th^ Aug 2017 (late winter), 16^th^ Mar 2018 (early autumn), 23^rd^ May 2018 (late autumn) and 19^th^ Sep 2018 (early spring). Newly eclosed adults were sexed and placed as a group of 30 to 40 flies in separate mesh cages and held under identical conditions as were flies of the captive cohort. These flies became the reference cohorts (R). Our methodology here differs from earlier studies using the demographic method through the use of multiple reference cohorts (previous studies use only one combined reference cohort created from flies collected at different time points), and through the grouping of individuals (previous studies held insects individually)^28^. We ran preliminary trials to test the effect of group (40 flies) and individual holding on fly survival and detected negligible differences, but grouping flies dramatically decreased the time spent in the insectary each day.

### Data collection

For captive cohorts, the day when wild flies were transferred to the insectary was considered as day zero. The fly cages were checked daily for any dead flies and their post-capture longevity recorded. For reference cohorts, the day when flies emerged was considered as day zero and longevity of the dead flies recorded by daily checking. The dead flies were removed from the cages regularly. If there were days when data was not recorded (an infrequent event), the number of dead flies was averaged over the missed day(s) to obtain daily mortality.

### Data analysis

#### Survival function of reference and captive flies

For an overall comparison of survival of captive and reference flies, the Kaplan–Meier survival function was run using combined data from all captive and reference cohorts (IBM SPSS Statistics 25). To compare the longevity of reference males from the five collections and females from the three collections following creation of the Kaplan–Meier survival functions, pairwise log-rank tests were conducted. For captive cohorts, pairwise log rank tests were conducted to compare the survival of the two captive cohorts collected during the same seasons in early autumn and early spring in 2017 and 2018.

#### Estimation of age-structure of wild flies

The data is sourced from observations of a captive cohort and a reference cohort. The captive cohort data is collected from a population of $W$ wild caught individuals—of unknown age—who were kept in captivity until they died. The length of time that elapses before each individual die is recorded in an $W$ element vector $\mathbf{V}$**.**

The reference cohort data was collected from a population of $R$ individuals that were born, raised, and died in captivity but originated from wild sources (infested fruits). The proportion of the cohort that are alive on day $t-1$, who die during day $t$ is recorded as element $t$ in vector $\mathbf{M}$, whose length is therefore as long as the oldest age (in days) observed in the reference individuals. We applied a 30-day moving-window average to the vector. Since the reference cohort was followed until the last individual dies at age $\omega$, the last element in $\mathbf{M}$ is equal to 1.

Our goal is to estimate a discrete probability density vector $\mathbf{A}$ that describes the age distribution of the captive cohort at the time of capture, and which provides a best-fit to the observed survival of that cohort. Given that the captive cohort could be composed of multiple sub-cohorts (i.e., multiple reproductive events), we do not assume a parametric form for this probability density function, which may be complicated and multimodal.

### Likelihood function

The log likelihood of observing the longevity vector $\mathbf{V}$, given a particular initial age distribution $\mathbf{A}$, is:

1 $$LL\left(\mathbf{V}|\mathbf{A}\right)=\sum_{i=1}^{W}\mathrm{ln}\left[\sum_{t=1}^{\omega }{\mathbf{A}}_{t}\mathbf{M}\left(t+{\mathbf{V}}_{i}\right)\prod_{\tau =t}^{t+{\mathbf{V}}_{i}-1}\left(1-\mathbf{M}\left(\tau \right)\right)\right]$$

The initial summation aggregates the independent contribution of each individual in the captive cohort to the likelihood. The second summation considers all possible ages of that individual at the time of capture, weighted by the probability that the individual survives for ${\mathbf{V}}_{i}$ days and then subsequently dies (according to the mortality rates observed in the reference cohort).

### Fitting the initial age distribution for the captive cohort

**Figure 4:**
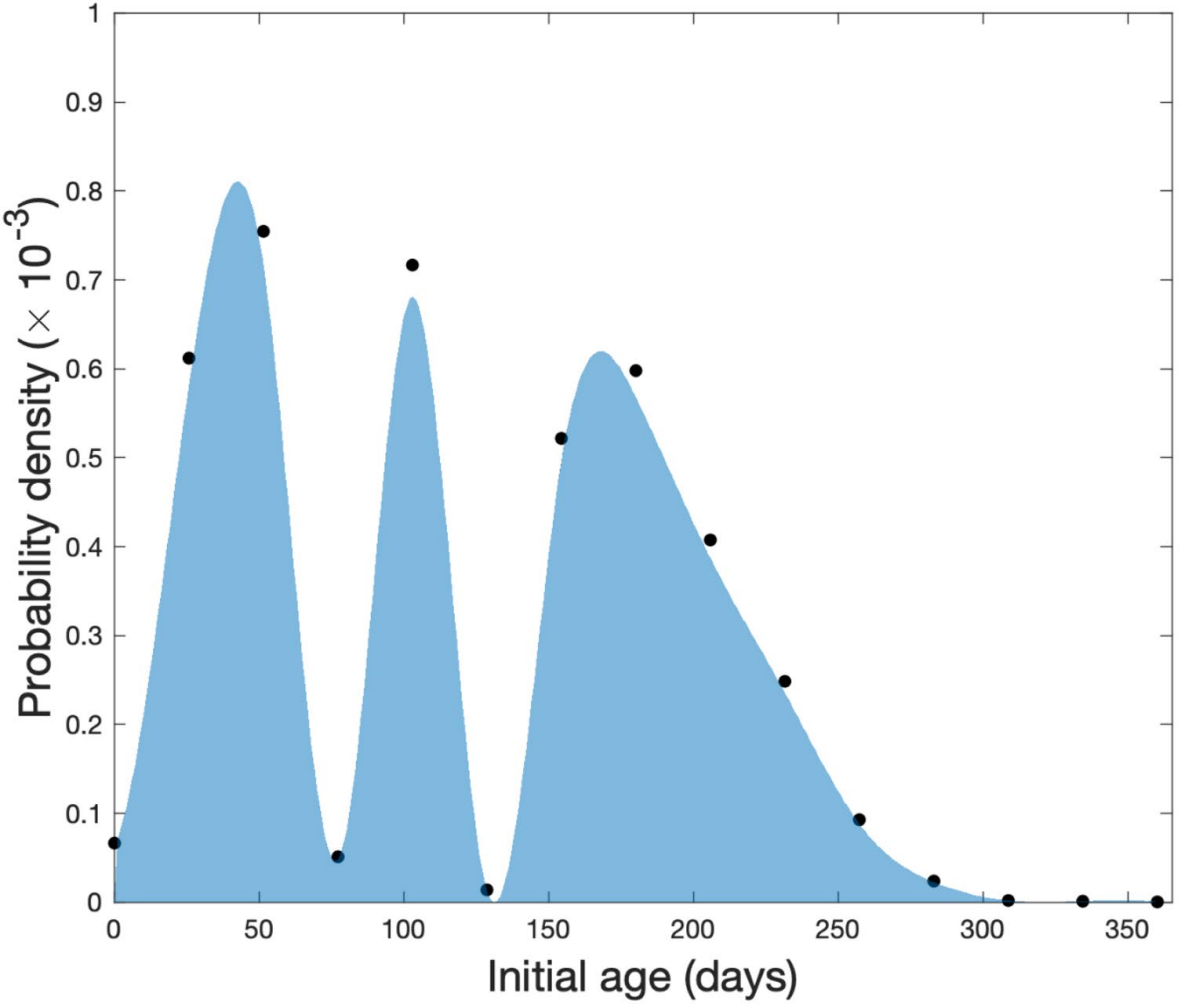
An example of age-distribution produced by maximum likelihood method using hypothetical data.

Thus, given an age distribution, we can calculate the likelihood of observing a given longevity vector in a captive cohort. The goal is therefore to efficiently search for the age distribution that maximizes the likelihood. We search for the best-fit distribution using nonparametric candidate functions for $\mathbf{A}$. Specifically, we fit a piecewise cubic spline to a set of 15 equidistant points between 1 and 365 days, whose values are initially chosen at random (Fig. 4). We calculate the likelihood of this proposed age distribution and vary the values of the 15 points using a constrained interior-point algorithm to minimize this measure. This algorithm is implemented in Matlab R2019a as the function *fmincon*. We repeat the search from several randomly selected initial guesses to ensure that our best-fit function is not a local maxima.

### Defining age-groups

Due to variation in the longevity of reference cohorts, attempts to age captive populations quantitatively gave variable results depending on the reference cohort used in model fitting. In contrast, when the modelled age-structure of a captive cohort is plotted against the survival curve of the utilised reference cohort, highly consistent qualitative ages could be estimated. Thus, we report the modelled age groups of captive cohorts as young, middle-age and old as relative measures depending on where the modelled age-distribution curve sits in relation to the survival curve of utilised reference cohort. “Young” populations are those for which the modelled population aligns with the survival curve where it is showing little or no population mortality, “middle-age” populations are those align with the survival curve where it is showing approximately 50% population mortality, and “old” populations are those align with the survival curve where it is showing approximately near 100% population mortality.

## Supplementary Information

Supplementary Information 1.

Supplementary Information 2.
