# Supplementary material for: A polyphagous, tropical insect herbivore shows strong seasonality in age-structure and longevity independent of temperature and host availability
Source: Sci Rep. 2021 Jun 1;11:11410. doi: 10.1038/s41598-021-90960-7 (PMC8169897; doi:10.1038/s41598-021-90960-7)
Supplement: Supplementary file 1 — Supplementary Information 1. [file 41598_2021_90960_MOESM1_ESM.docx]

**Choosing the reference cohort (AICw)**

As longevity of reference flies showed strong seasonality (a highly unexpected result, see later), instead of combining the data from reference cohorts collected during various seasons (Carey et al. 2008, Carey et al. 2012), a single season’s reference cohort was paired with a captive cohort selected based on maximum likelihood ratio and AIC weight that gave the best fitted age-distribution for the captive cohort.

We calculated the best-fit age distribution for each observed captive cohort, using each available reference cohort in turn. Because there are eight potential reference cohorts (i.e. reference cohorts collected during five seasons, a combined reference cohort from the same season replications collected in late autumn 2017 and 2018, a combined reference cohort from two close seasons [late winter and early spring], and the combination of all five seasons) this results in eight best-fit distributions, and eight maximum likelihood values. We used Akaike Information Criteria weights (AICw) to determine which of these reference cohorts best explained the observed mortality dynamics. The AICw is effectively proportional to the probability that each model (reference cohort) minimizes the information lost by using that model to represent the truth:

$$\mathrm{AICw}_{i}=\exp\left[ \frac{\mathrm{AIC}_{\min}-\mathrm{AI}C_{i}}{2} \right]$$

where $\mathrm{AIC}_{i}$ is calculated from the maximum $LL$ (see Eq. 1) for reference cohort $i$.
