# Supplementary material for: A polyphagous, tropical insect herbivore shows strong seasonality in age-structure and longevity independent of temperature and host availability
Source: Sci Rep. 2021 Jun 1;11:11410. doi: 10.1038/s41598-021-90960-7 (PMC8169897; doi:10.1038/s41598-021-90960-7)
Supplement: Supplementary file 2 — Supplementary Information 2. [file 41598_2021_90960_MOESM2_ESM.docx]

Supplementary Table 1. Akaike Information Criteria Weight (AICw) calculated from maximum likelihood for eight potential reference cohorts against each season’s captive cohort. The best-fitted age-distribution for a season were chosen from maximum AICw value which are indicated by bold numbers.

| **Captive Cohorts** | **Reference cohorts** | | | | | | | |
| --- | --- | --- | --- | --- | --- | --- | --- | --- |
| Seasons  and dates | Late autumn 24/05/17 | Late winter 17/08/17 | Early autumn 16/03/18 | Late autumn 23/05/18 | Early spring 19/09/18 | Late autumn  2017-18 | Late winter 2017 + Early spring 2018 | All seasons |
| Early autumn 13/03/17 | 0.006 | 0.029 | 0.002 | **0.803** | 3.13E-123 | 0.130 | 0.017 | 0.013 |
| Early autumn 13/03/17 | 0.005 | 0.009 | **0.493** | 0.446 | 8.75E-85 | 0.034 | 0.009 | 0.003 |
| Late winter  17/08/17 | 8.9E-41 | **0.560** | 1.19E-38 | 7.44E-42 | 9.75E-79 | 1.77E-40 | 0.281 | 0.158 |
| Early spring  7/09/17 | 0.089 | 0.178 | 0.133 | 0.025 | 0.013 | **0.261** | 0.183 | 0.117 |
| Early summer 17/11/17 | 0.044 | 0.119 | 0.082 | **0.467** | 1.04E-161 | 0.135 | 0.092 | 0.062 |
| Early autumn 16/03/18 | 2.81E-19 | 1.55E-17 | 4.35E-17 | **1** | 1.73E-50 | 3.87E-08 | 7.12E-18 | 3.21E-22 |
| Early spring 19/09/18 | 0.062 | 0.208 | 0.205 | 0.074 | 3.53E-41 | 0.084 | **0.217** | 0.150 |
